# Genome Sequences of Bacillus sporothermodurans Strains Isolated from Ultra-High-Temperature Milk

**DOI:** 10.1128/MRA.00145-19

**Published:** 2019-05-30

**Authors:** Rodney Owusu-Darko, Mushal Allam, Sílvia D. de Oliveira, Carlos A. S. Ferreira, Sunita Grover, Senzo Mtshali, Arshad Ismail, Rashmi H. Mallappa, Frederick Tabit, Elna M. Buys

**Affiliations:** a Department of Consumer and Food Sciences, University of Pretoria, Pretoria, South Africa; b National Institute for Communicable Diseases, Sandringham, South Africa; c School of Sciences, Pontifícia Universidade Católica do Rio Grande do Sul, Porto Alegre, Brazil; d Dairy Microbiology Division, Molecular Biology Unit, National Dairy Research Institute, Karnal, India; e Department of Life and Consumer Sciences, University of South Africa, Pretoria, South Africa; University of Arizona

## Abstract

Here, we report the draft genome sequences of 3 Bacillus sporothermodurans strains isolated from ultra-high-temperature milk products in South Africa and Brazil and the type strain MB 581 (DSM 10599). The genomes will provide valuable information on the molecular dynamics of heat resistance in *B*. *sporothermodurans*.

## Abstract (precis)

Here, we report the draft genome sequences of 3 Bacillus sporothermodurans strains isolated from ultra-high-temperature milk products in South Africa and Brazil and the type strain MB 581 (DSM 10599). The genomes will provide valuable information on the molecular dynamics of heat resistance in *B*.

## ANNOUNCEMENT

Bacillus sporothermodurans is a thermoresistant Gram-positive bacterium that can produce highly heat-resistant endospores (HRS) capable of surviving ultra-high-temperature (UHT) heat treatments (1, 2). First detected in UHT milk (3), it has subsequently been isolated from other dairy products, including UHT cream, chocolate milk, evaporated milk, and reconstituted milk (4). Furthermore, *B. sporothermodurans* has been isolated from non-dairy-based foods, including Indian curry (5), as well as from marine sources (6). After heat processing, the surviving spores may germinate and grow in the stored milk (1). The spores of *B. sporothermodurans* grow at low levels (≈10^5^ CFU/ml) and do not affect the pH of the milk (2); as a result, its presence may go unnoticed. However, there are reports of *B. sporothermodurans* strains isolated in Brazil causing significant proteolytic activity leading to UHT milk spoilage (7). If spoilage does occur, though, there may be slight changes in color, off flavors, and the destabilization of casein micelles (2). Consequently, the main concerns to the dairy industry are milk quality, nonsterility of milk products, and biofilm formation in milk processing equipment.

*B. sporothermodurans* strains SAD and SA01 were isolated from UHT milk produced in South Africa, and *B. sporothermodurans* strain BR12 was isolated from UHT milk from Brazil. The type strain *B. sporothermodurans* DSM 10599 was obtained from Leibniz Institute DSMZ-German Collection of Microorganisms and Cell Cultures GmbH (DSMZ). The enumeration and isolation of strains SAD and SA01 were undertaken as previously described (8), with the substitution of plate count agar for brain heart infusion (BHI) agar (Oxoid, UK). Single colonies of overnight fresh cultures of all four strains of *B. sporothermodurans* were inoculated into BHI broth (Oxoid) and incubated at 37°C for 72 hours. Genomic DNA was extracted using the ZR bacterial DNA miniprep kit (Zymo Research, USA) and quantified using the Qubit instrument and double-stranded DNA (dsDNA) broad-range (BR) assay kit (Life Technologies, USA). Multiplexed paired-end libraries were prepared using the Nextera XT DNA sample preparation kit (Illumina, USA). Genome sequencing was carried out on an Illumina MiSeq system. The paired-end reads (2 × 300 bp) were checked for quality and trimmed and *de novo* assembled using the CLC genomics workbench version 9 (Qiagen, Netherlands), with all low-quality (Q, <20) data filtered out. The resultant contigs were submitted to GenBank, where gene annotation was implemented using the NCBI Prokaryotic Genome Annotation Pipeline (PGAP) (9). The annotation was further uploaded to Rapid Annotation using Subsystem Technology (RAST) for subsystems‐based annotation (10–12).

The four assembled genomes had an average G+C content of 38.6%. Overall, genomes of *B. sporothermodurans* contain heat shock and hyperosmotic proteins (including DnaJ, GrpE, and GroEL) that will have an influence on the heat resistance and, consequently, the processing dynamics of food products. Additionally, all four strains sequenced contain the biofilm matrix protein component TasA and its homologs, which have been shown to be the major biofilm matrix component (13), especially in Bacillus subtilis. Ultimately, the whole-genome sequence of *B*. *sporothermodurans* will help improve our understanding of the heat resistance of this bacterium with the view of improving milk quality.

### Data availability.

The genome sequences of all four strains of *B. sporothermodurans* are publicly available at NCBI GenBank under the BioProject accession number PRJNA379529. Raw and trimmed sequencing reads have been deposited in the NCBI SRA under the study accession number SRP188520. The GenBank and SRA accession numbers are listed in Table 1. This announcement represents the first version of all four genomes.

**TABLE 1:** Summary report of the *de novo* assembly of four *B. sporothermodurans* strains

Organism	GenBank accession no.	SRA accession no.	Total no. of reads	Genome size (bp)	No. of coding sequences	Coverage (×)	No. of contigs	*N*_50_ (kpb)
*B. sporothermodurans* DSM 10599	NAZD00000000	SRR8741694	3,570,064	3,783,858	4,257	226	527	15,402
*B. sporothermodurans* SAD	NAZB00000000	SRR8732968	2,523,238	3,857,089	4,111	175	110	114,649
*B. sporothermodurans* SA01	NAZA00000000	SRR8732969	1,858,252	3,414,010	3,768	146	290	22,386
*B. sporothermodurans* BR12	NAZC00000000	SRR8741693	3,421,418	3,974,872	4,558	193	805	9,377
